# Internet-Based Physical Activity Interventions: A Systematic Review of the Literature

**DOI:** 10.2196/jmir.9.3.e26

**Published:** 2007-09-30

**Authors:** Marleen H van den Berg, Johannes W Schoones, Theodora PM Vliet Vlieland

**Affiliations:** ^2^Walaeus LibraryLUMCLeidenThe Netherlands; ^1^Department of RheumatologyLeiden University Medical Center (LUMC)LeidenThe Netherlands

**Keywords:** Physical activity, exercise, Internet, behaviour change, systematic review, consumer health informatics

## Abstract

**Background:**

Nowadays people are extensively encouraged to become more physically active. The Internet has been brought forward as an effective tool to change physical activity behavior. However, little is known about the evidence regarding such Internet-based interventions.

**Objective:**

The aim of the study was to systematically assess the methodological quality and the effectiveness of interventions designed to promote physical activity by means of the Internet as evaluated by randomized controlled trials.

**Methods:**

A literature search was conducted up to July 2006 using the databases PubMed, Web of Science, EMBASE, PsycINFO, and Cochrane Library. Only randomized controlled trials describing the effectiveness of an Internet-based intervention, with the promotion of physical activity among adults being one of its major goals, were included. Data extracted included source and year of publication, country of origin, targeted health behaviors, participants’ characteristics, characteristics of the intervention, and effectiveness data. In addition, the methodological quality was assessed.

**Results:**

The literature search resulted in 10 eligible studies of which five met at least nine out of 13 general methodological criteria. The majority of the interventions were tailored to the characteristics of the participants and used interactive self-monitoring and feedback tools. Six studies used one or more theoretical models to compose the contents of the interventions. One study used an objective measure to assess the amount of physical activity (activity monitor), and six studies used multiple subjective measures of physical activity. Furthermore, half of the studies employed measures of physical fitness other than physical activity. In three studies, an Internet-based physical activity intervention was compared with a waiting list group. Of these three studies, two reported a significantly greater improvement in physical activity levels in the Internet-based intervention than in the control group. Seven studies compared two types of Internet-based physical activity interventions in which the main difference was either the intensity of contact between the participants and supervisors (4 studies) or the type of treatment procedures applied (3 studies). In one of these studies, a significant effect in favor of an intervention with more supervisor contact was seen.

**Conclusions:**

There is indicative evidence that Internet-based physical activity interventions are more effective than a waiting list strategy. The added value of specific components of Internet-based physical activity interventions such as increased supervisor contact, tailored information, or theoretical fidelity remains to be established. Methodological quality as well as the type of physical activity outcome measure varied, stressing the need for standardization of these measures.

## Introduction

Regular physical activity is associated with lower morbidity and mortality rates from cardiovascular disease [1-4], diabetes mellitus [5], cancer [6], and osteoporosis [7]. Despite these proven health benefits, the majority of the adult population in Western nations does not meet the public health recommendations for physical activity [8-12]. Therefore, there is a need for the delivery of effective interventions aimed at positively influencing physical activity behavior.

Traditionally, most physical activity interventions use face-to-face modes of delivery (eg, individual consultations or group meetings). Their mainly short-term effectiveness has been extensively documented in a number of systematic reviews [13-18]. In addition, these reviews demonstrated that many of the physical activity studies suffer from several methodological weaknesses. The main methodological shortcomings identified by these reviews included use of physical activity measures without validity/reliability data, exclusive reliance on self-report measures, inadequate control of confounding factors, small sample sizes, lack of data on follow-up, and low follow-up rates.

With the number of people having access to and using the Internet rapidly increasing [19], the Internet is more and more used as a mode of delivery for physical activity programs. The strength of Internet-based physical activity interventions lies in the fact that with this mode of delivery large numbers of individuals can be reached at lower costs than with face-to-face interventions [20]. Moreover, by using the Internet, participants can access large amounts of information, and they can choose the time when they would like to interact and receive information [21].Previous reviews on the effectivenessof Web-based physical activity interventions have indicated that the Internet can indeed serve as a promising mode of delivering physical activity interventions [20-24]. However, most of these reviews need to be updated as they comprised studies that were conducted between 2000 and 2003. This is all the more important as previous reviews included mainly observational and anecdotal studies, whereas a number randomized controlled trials have been published over recent years. Moreover, specific methodological characteristics of studies on physical activity interventions, such as the measurement of physical activity, have not yet been addressed in reviews that were exclusively aimed at Internet-based interventions.

The aim of this review is therefore to systematically assess both the methodological quality and the effectiveness of interventions designed to promote physical activity by means of the Internet as evaluated by randomized controlled trials.

## Methods

### Definitions

Physical activity and exercise represent different concepts: physical activity is defined as any bodily movement resulting in energy expenditure; exercise is a subset of physical activity that is planned, structured, repetitive, and aimed at improving or maintaining physical fitness [25]. Since exercise falls under the broader concept of physical activity, in this paper we will use the term physical activity.

In addition, since email communication is based on Internet technology, both the use of websites and email will be designated as an Internet-based intervention.

### Search Strategy

In cooperation with a trained librarian (JS), a search strategy was composed. The following databases were searched: PubMed (1949 to July 2006), Web of Science (1945 to July 2006), EMBASE (OVID-version, 1980 to July 2006), PsycINFO (1887 to July 2006), and Cochrane Library (1990 to July 2006). The search strategy consisted of the AND combination of three main concepts: Internet, physical activity, and intervention. For these three concepts, all relevant keyword variations were used, not only keyword variations in the controlled vocabularies of the various databases, but the free text word variations of these concepts as well. In general, the search consisted of the combination of the following terms: (1) internet or worldwideweb or world wide web or information technology or cyber* or web or website* or interactive or email or e-mail or e mail or emails or e-mails or e mails or emailing or e-mailing or e mailing or electronic mail; (2) physical education and training or exercise therapy or physical fitness or exercise or motor activity or physical training or physical education or fitness or exercise* or physical activity or physical activities or physical inactivity; and (3) intervention or interventions or intervention* or treatment outcome or intervention studies or epidemiologic study characteristics or study characteristics or epidemiologic methods or program or programs or programme or programmes or programmed or program evaluation.

This search strategy was optimized for all consulted databases, taking into account the differences of the various controlled vocabularies as well as the differences of database-specific technical variations (eg, different truncation symbols). Details of the database searches can be obtained from the author.

### Selection of Articles

To be included, articles had to describe an intervention in which one of the primary goals was the promotion of physical activity among adults (18 years or older). Furthermore, the intervention had to be delivered predominantly by means of the Internet in one of the following ways: (1) exchange of information via the World Wide Web between a health care setting and an individual (eg, between a clinic and a participant’s home or workplace), (2) use of email for communication between a therapist or health care professional and a patient (or patient group). Internet-based physical activity interventions that promoted physical activity in order to achieve a secondary goal, such as weight reduction, were also included.

Only randomized controlled trails with pretest and posttest outcome data for both the control and intervention groups were considered for inclusion in this review. No restrictions were defined regarding the type and contents of the control group: this could be assignment to a waiting list, a non-Internet-based intervention, or a different type of Internet-based intervention. At least one of the outcomes had to be described in terms of change in physical activity level (eg, change in amount or quantity of physical activity). Furthermore, because of limited resources for translation, this review was restricted to publications in English, Dutch, and German.

The reference lists of the selected articles were checked for additional eligible articles, using the same inclusion criteria. Review articles could not be included in the review; however, the reference lists of relevant review articles were also checked for additional eligible articles. The articles were independently selected and assessed by two reviewers (MvdB and TVV).

### Assessment of Methodological Quality

With respect to the guidelines for evaluating methodological quality of intervention studies, the literature does not provide a gold standard. We used a list of criteria recommended by Van Tulder et al [26], which has proven to be appropriate in other reviews evaluating physical activity or exercise interventions [27,28]. This list was based on the guidelines for systematic reviews as set by the editorial board of the Cochrane Collaboration Back Review Group, which address the main steps in conducting a systematic review: literature search, inclusion criteria, methodological quality, data extraction, and data analysis.The list of Van Tulder et al contains 19 methodological criteria. The criteria “care provider blinded,” “patient blinded,” “co-interventions avoided,” and “description of adverse effects” were not regarded as being suitable or relevant by the reviewers because of the character of the interventions and were removed from the list. The criteria “relevant outcome measures” and “short-term follow-up outcome” were already used as inclusion criteria for articles in this review; therefore, these criteria were not used for assessing methodological quality. Finally, the criterion “acceptable compliance” was reformulated as “description of compliance,” and “description of and acceptable dropout rate” was reformulated as “description of dropout rate plus comparison of dropouts with completers.” The final number of criteria used to assess methodological quality was 13 (see the Multimedia Appendix). All criteria were scored as “yes,” “no,” or “unclear.” Equal weight was applied to all criteria, resulting in a methodological summary score ranging from 0 to 13. The literature provides no guidelines for choosing cutoff points in order to rate the methodological quality [29]. In this review, we rated the studies as having good methodological quality if two thirds or more of the criteria were met (ie, a summary score of 9 or higher).

In addition, we evaluated the studies included in this review with respect to quality criteria that apply to physical activity interventions and Internet-based interventions in particular. These criteria were derived from previous literature on physical activity assessment in general [30] and on evaluation methods of Internet-based behavioral interventions [31,32] and comprised the following:

- Intervention-related: (1) tailoring of program to participants’ characteristics, (2) use of interactive self-monitoring and feedback, (3) theoretical fidelity (degree to which interventions follow their planned procedures or theoretical models)
- Process-related: (4) information on use of intervention tools or facilities
- Outcome-related: (5) use of a combination of physical activity measurements (rather than one measure), (6) use of objective methods of data collection, such as activity monitors, heart rate monitors, pedometers, direct observation, or doubly labelled water, (7) use of additional fitness-related outcomes

All quality criteria were scored as “yes,” “no,” or “unclear.”

### Data Extraction

This review is a qualitative systematic review as the data extracted from the selected studies were summarized but not statistically combined. Aggregating findings across studies rather than pooling them was a more useful method of describing synthesis, as the outcome measures varied widely. The results of the selected studies were broken down, thoroughly analyzed, and then combined into a whole via a listing of themes. This has proven to be a suitable method for systematic reviews [33].

The following information was systematically extracted by the two reviewers: source and year of publication, country of origin, targeted health behaviors (physical activity, weight loss, nutrition behavior, or other), characteristics of the study population (number and type of participants, age, gender), characteristics of the intervention (duration, theoretical foundation, description of contents), and pretest and posttest physical activity outcomes of both intervention groups. With respect to the changes in physical activity level, only the posttest results measured directly after finishing the physical activity intervention were included.

In order to be able to make more valid comparisons, the selected studies are divided into three categories: section A contains studies in which Internet-based physical activity interventions were compared with a waiting list or an attention-control group; section B contains studies in which two types of Internet-based physical activity interventions were compared that mainly differed with respect to the amount or frequency of contact between the participants and supervisors; in section C, two types of Internet-based physical activity interventions were compared; however, in these studies, the two interventions varied with respect to the applied treatment procedures.

Reviewers were blinded to authorship, journal title, and other study-related information. Furthermore, screening for eligible articles as well as data extraction from the selected articles were done independently. Any discrepancies between the two reviewers were settled by consensus.

## Results

### Selection of Articles

**Figure 1:**
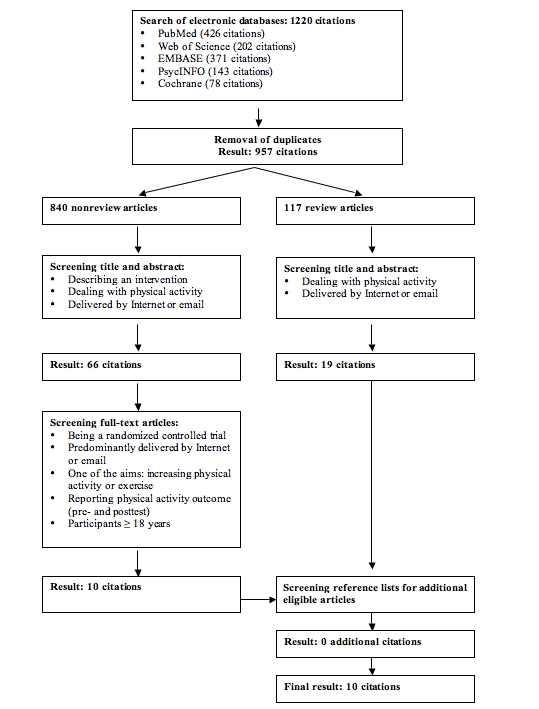
Article search and selection process

Figure 1 illustrates the search and selection process. The initial database search yielded 1220 citations. After eliminating duplicates, this was reduced to 957 citations, of which 117 were review articles. Screening titles and abstracts of the 840 nonreview articles resulted in 66 citations potentially meeting eligibility criteria. After completely reviewing the corresponding full-text articles, the total number of articles was reduced to 10. Reasons for exclusion of the other 56 citations were not reporting pretest and posttest physical activity outcomes (n = 25), intervention not predominantly delivered by the Internet (n = 16), not being a randomized controlled trial (n = 13), and participants being younger than 18 years (n = 2). Screening the titles and abstracts of the 117 review articles resulted in 19 relevant reviews. Screening both the reference list of these reviews, as well as the reference lists of the 10 selected articles, did not bring up any additional articles. As a result, 10 articles were included.

### Assessment of Methodological Quality

Results of the methodological assessment are described in Table 1. Five studies met nine or more criteria [32,34-37], implying a good methodological quality. One study described the method of random assignment and stated that this assignment was performed by an independent person [37]. Information about the blinding of the outcome assessor was given in two studies [34,37]. None of the studies performed a full intention-to-treat analysis according to the definition of intention-to-treat given by Hollis and Campbell [38], stating that “a full application of intention-to-treat is possible only when complete outcome data are available for all randomised subjects.” All studies reported a dropout rate, with six of the 10 studies comparing the characteristics of these dropouts with the subjects that completed all outcome measurements [32,35,36,39-41]. In two studies [41,42], the study sample included only those participants who completed both the baseline as well as the follow-up measurements, excluding dropouts from the analysis.

**Table 1:** Methodological quality of the studies

	Kosma et al [41]	Plotnikoff et al [42]	Napolitano et al [39]	Marshall et al [34]	Van den Berg et al [37]	Hageman et al [43]	Rovniak et al [32]	Tate et al [36]	McKay et al [40]	Tate et al [35]
Specification of eligibility criteria	yes	yes	yes	yes	yes	yes	yes	yes	yes	yes
Description of randomization method	no	no	no	yes	yes	no	yes	yes	no	no
Random assignment performed by independent person	unclear	unclear	unclear	unclear	yes	unclear	unclear	unclear	unclear	unclear
Groups similar at baseline	yes	yes	yes	yes	yes	yes	yes	yes	yes	yes
Sufficient description of interventions	yes	yes	yes	yes	yes	yes	yes	yes	yes	yes
Description of compliance with interventions	no	no	yes	yes	yes	yes	yes	yes	yes	yes
Blinding of outcome assessor	unclear	unclear	unclear	yes	yes	unclear	no	unclear	unclear	unclear
Description of dropout rate plus comparison of dropouts and completes	yes	no	yes	no	no	no	yes	yes	yes	yes
Outcome assessment ≥ 6 months after randomization	no	no	no	no	yes	no	yes	yes	no	yes
Timing of assessments comparable	yes	yes	yes	yes	yes	yes	yes	yes	yes	yes
Description of sample size calculation	no	no	no	yes	yes	yes	no	no	no	yes
Intention-to-treat analysis	no	no	no	no	no	no	no	no	no	no
Presentation of point estimates and variability measures	yes	yes	yes	yes	yes	yes	yes	yes	yes	yes
**Total number of criteria fulfilled**	**6**	**5**	**7**	**9**	**11**	**7**	**9**	**9**	**7**	**9**

Concerning the quality criteria that apply to physical activity interventions and Internet-based interventions in particular, the results show that in the majority of the studies the interventions were tailored to the characteristics of the participants and used interactive self-monitoring and feedback tools (Table 2). Six studies used one or more theoretical models to compose the information delivered to the intervention group [39-42] or to both the intervention and control groups [32,34]. These models were the Transtheoretical Model [34,39,41,42], the Protection Motivation Theory [42], the Theory of Planned Behavior [42], the Social Cognitive Theory [32,39,42], and a social-ecological model [40].

With respect to outcome measurement, one study [37] used an objective measure to assess the amount of physical activity (activity monitor), and six studies used multiple subjective measures of physical activity [34,37,39,40,42,43]. Half of the studies employed measures of physical fitness other than physical activity [32,35-37,43].

**Table 2:** Characteristics of intervention, process, and outcome measures of the studies

	Kosma et al [41]	Plotnikoff et al [42]	Napolitano et al [39]	Marshall et al [34]	Van den Berg et al [37]	Hageman et al [43]	Rovniak et al [32]	Tate et al [36]	McKay et al [40]	Tate et al [35]
**Intervention**										
Program tailored to participants’ characteristics	no	no	yes	yes	yes	yes	yes	yes	yes	yes
Use of interactive self-monitoring and feedback	no	no	yes	yes	yes	no	yes	yes	yes	yes
Intervention developed according to theoretical guidelines	yes	yes	yes	yes	no	no	yes	no	yes	no
**Process**										
Use of intervention tools/facilities	no	no	no	yes	yes	yes	yes	yes	yes	yes
**Outcome**										
Use of combination of physical activity assessment measures	no	yes	yes	no	yes	yes	no	no	no	no
Use of objective physical activity assessment methods	no	no	no	no	yes	no	no	no	no	no
Use of additional physical fitness–related outcomes	no	no	no	no	yes	yes	yes	yes	no	yes

### Data Extraction

#### Characteristics of Selected Studies

Study characteristics are described in Table 3. Seven of the 10 selected studies were performed in the United States, one in Canada, one in Australia, and one in The Netherlands. All studies were published between 2001 and 2006. Three studies addressed interventions targeted at both physical activity and nutrition behavior; the other seven studies focused on interventions aimed at physical activity behavior only. The duration of the interventions varied from 1 to 12 months, with three studies describing interventions of 6 months or longer [35-37].

**Table 3:** Characteristics of studies and participants*

Study	Targeted Health Behavior	Duration of Intervention (months)	Sample Description				
			No. of Participants Randomized	No. of Participants With Complete Data	Type of Participants	Gender (% male)	Age (mean ± SD; years)

Kosma et al [41], 2005, USA	PA	1	151 (I: 101, C: 50)	75 (I: 46, C: 29)	Inactive adults with physical disabilities, with Internet access	21	38.7 ± 8.9
Plotnikoff et al [42], 2005, Canada	PA and nutrition behavior	3	2598 (I: ?, C: ?)	2121 (I: 1566, C: 555)	Employees of large workplaces with Internet and email access	26	44.9 ± 6.3
Napolitano et al [39], 2003, USA	PA	3	65 (I: 30, C: 35)	52 (I: 21, C: 31)	Hospital employees participating in ≤ 120 min of moderate PA/week or ≤ 60 min of vigorous PA/week, with Internet and email access	14	42.8 ± 10.0
Marshall et al [34], 2003, Australia	PA	2	655 (I: 327, C: 328)	512 (I: 250, C: 262)	University employees with email access	49	43 ± 11
Van den Berg et al [37], 2006, The Netherlands	PA	12	160 (I: 82, C: 78)	152 (I: 77, C: 75)	Patients with rheumatoid arthritis not participating in 30 min of moderate PA on ≥ 5 days/week, with Internet and email access	24	49.6 ± 10.3
Hageman et al [43], 2005, USA	PA	2	31 (I: 15, C: 16)	30 (I: ?, C: ?)	Healthy women not participating in 30 min of moderate PA on ≥ 5 days/week, with Internet access	0	56.1 ± 4.9
Rovniak et al [32], 2005, USA	PA	3	61 (I: 30, C: 31)	50 (I: 25, C: 25)	Sedentary adult women participating in < 90 min of PA/week, with email access	0	40.2 ± 9.1
Tate et al [36], 2003, USA	Weight loss (PA and nutrition)	12	92 (I: 46, C: 46)	77 (I: 38, C: 39)	Overweight (BMI 27-40 kg/m^2^) adults at risk of type 2 diabetes, with Internet and email access	10	48.5 ± 9.4
McKay et al [40], 2001, USA	PA	2	78 (I: 38, C: 40)	68 (I: 35, C: 33)	Type 2 diabetic patients not participating in 30 min of moderate PA on ≥ 5 days/week, with Internet and email access	47	52.3 ± ?
Tate et al [35], 2001, USA	Weight loss (PA and nutrition)	6	91 (I: 46, C: 45)	71 (I: 36, C: 35)	Overweight (BMI 25-36 kg/m^2^) adult hospital employees with Internet and email access	11	40.9 ± 10.6

^*^PA indicates physical activity; I, intervention group; C, control group; ?, unknown; BMI, body mass index.

#### Characteristics of Study Populations

Table 3 shows that the total population size varied from 31 to 2598 participants. The study populations all consisted of healthy (overweight) adults, except for the studies of Kosma et al [41], McKay et al [40], and Van den Berg et al [37], which included physically disabled patients, diabetic patients, and patients with rheumatoid arthritis, respectively. Six of the 10 studies were specifically targeted at adults who were sedentary at baseline [32,37,39-41,43]; the other four studies did not employ any inclusion criteria regarding baseline physical activity level [34-36,42]. In two studies [34,40], the proportion of male and female participants was almost equal; in the other studies, the large majority of participants were female. Mean age varied from 39 to 56 years.

#### Characteristics of the Interventions

Table 4 describes the characteristics of the Internet-based physical activity programs and control conditions. 

Section A of Table 4 describes the three studies in which an Internet-based physical activity intervention was compared a waiting list group [39,41,42]. In two of these studies [39,41], the participants in the Internet-based intervention had access to a website and received emails; in the other study, the intervention group received only emails [42].

Section B of Table 4 describes the four studies that compared two types of Internet-based physical activity intervention with the main difference being the amount or frequency of contact between the participants and supervisors [35-37,40]. These studies investigated whether more intensive supervision would lead to a greater increase in physical activity level. In three studies, the difference in the amount of supervisor contact was, in fact, a difference in the degree of tailoring or personalization [35,37,40], in which participants from the intervention group had access to a website and received emails, whereas the control group had website access only. In the other study [36], website access and email communication was offered to participants in both the intervention and control groups.

Section C of Table 4 describes the three studies that compared two types of Internet-based physical activity intervention in which the main difference was the treatment procedures that were used, whereas the amount of contact between the participants and supervisors did not differ. One study [32] investigated whether precision in replicating theory-based recommendations influenced the effectiveness of an Internet-based physical activity intervention. In the second study [34], the means by which the physical activity interventions were delivered differed (print-based versus Web-based). The third study was designed to explore the net effect of tailored versus standard information.

Eight studies aimed to increase any type of physical activity, whereas two studies were specifically targeted at walking [32] or cycling on a bicycle ergometer [37].

#### Effectiveness of Intervention

The physical activity outcome measures of both the intervention and control groups are expressed as pretest and posttest results and are described in Table 4. Four studies included one physical activity outcome parameter [32,35,36,41], five studies included two physical activity parameters [34,39,40,42,43], and one study reported more than two physical activity parameters [37]. Five of the 10 selected studies reported additional physical fitness–related outcome measures such as cardiorespiratory fitness, flexibility, and body weight [32,35-37,43]. In three of these five studies [32,35,36], the reported changes in physical activity level were considered a secondary outcome; primary outcomes in these studies were changes in body weight and waist circumference [35,36], cardiorespiratory fitness, and walking speed [32].

Regarding the four studies described in Section A of Table 4, in which Internet-based interventions were compared with a waiting list, two studies reported significant differences between the intervention and control groups [39,42]. With respect to the four studies described in Section B of Table 4, in which the intensity of contact in two types of Internet-based physical activity intervention varied, one study reported significant differences between the intervention and control groups with respect to change in physical activity level [37]. Two of the four studies [35,36] in Section B were not primarily aimed at increasing physical activity level, but rather to decrease body weight and waist circumference.

The changes in physical activity level were all nonsignificant in the three studies in which the applied treatment procedures of two Internet-based physical activity interventions varied (Table 4, Section C). This section comprised one study in which physical activity was not the primary outcome measure [32].

**Table 4:** Characteristics and results of the Internet-based physical activity interventions*

Study	Description of Intervention Group	Description of Control Group	PA Outcome Measures^†^			Additional Fitness-Related Outcomes	Conclusion
			Type of PA Outcome Variable	PA Pre-test Results (mean ± SD)	PA Post-test Results (mean ± SD)		

**Section A: comparison of an Internet-based physical activity intervention with a waiting list or attention-control group**							
Kosma et al [41], 2005	Weekly emails containing a Web link to motivational PA lesson plans; opportunity to participate in Web-based discussion board, for half of intervention group	Weekly emails containing messages not related to PA	Leisure time PA (MET hours/day)	I: 6.1 ± 7.4 C: 9.3 ± 7.7	I: 8.2 ± 6.8 C: 6.9 ± 7.8	–	No significant between-group differences for leisure time PA
Plotnikoff et al [42], 2005	Weekly emails containing PA information operationalizing social-cognitive items and beliefs predicting PA behavior and links to other websites about PA and healthy eating	No weekly emails (nothing)	Moderate and vigorous PA (MET min/week) Workplace activity status (1 = sedentary to 4 = very active)	PA: I: 664.1 ± 726.1 C: 668.6 ± 752.6 Workplace status: I: 1.3 ± 0.6 C: 1.3 ± 0.5	PA: I: 683.7 ± 702.3 C: 592.7 ± 652.8 Workplace status: I: 1.4 ± 0.6 C: 1.4 ± 0.6	–	Significant between-group differences for moderate and vigorous PA, not for workplace status
Napolitano et al [39], 2003	Access to stage-based PA website containing the following sections: activity quiz, safety tips, becoming active, PA and health, overcoming barriers, planning PA, and benefits of PA Weekly tip sheets sent by email containing PA-related information about getting started, monitoring progress, setting goals, rewarding, and support Opportunity to contact helpline by email or telephone in case of questions, concerns, or problems	Waiting list	Moderate intensity PA (min/week) Walking (min/week)	Moderate PA: I: 68.8 ± 58.1 C: 80.9 ± 77.8 Walking: I: 57.2 ± 56.9 C: 87.6 ± 177.4	Moderate PA: I: 112.0 ± 75.7 C: 82.0 ± 87.3 Walking: I: 99.8 ± 68.3 C: 68.4 ± 85.2	–	Significant between-group differences for moderate intensity PA and walking
**Section B: comparison of two types of Internet-based physical activity interventions that differ with respect to amount of contact between the participants and supervisors**							
Van den Berg et al [37], 2006	Access to website containing a personalized PA program consisting of weekly personalized physical activity schedules with weekly personalized feedback provided by physical therapist Access to online discussion forum to contact other participants Access to face-to-face group meetings very 3 months A bicycle ergometer was given on loan during intervention period	Access to website containing general PA information, which was updated once a month Opportunity to order free copy of PA-related CD-ROM	Moderate PA (% patients meeting moderate PA recommendations) Vigorous PA (% patients meeting vigorous PA recommendations)	Moderate proportions: I: 0 C: 0 Vigorous proportions: I: 6 C: 1	Moderate %: I: 26 C: 15 Vigorous %: I: 34 C: 10	Functional ability	Significant between-group differences for vigorous PA, not for moderate PA
Tate et al [36], 2003	One introductory face-to-face group weight loss session (1 hour) in which instructions regarding weight loss and increasing PA levels were given by clinical therapist Access to educational website containing information about weight loss, including tips, links, and other Internet resources Instructions to report dietary and PA self-monitoring information weekly by means of website diary 5 emails per week sent by therapist in the first month, weekly emails for remaining 11 months; emails contained personalized feedback, recommendations, reinforcements, answers to participants’ questions, and general support	One introductory face-to-face group weight loss session (1 hour) in which instructions regarding weight loss and increasing PA levels were given by clinical therapist Access to educational website containing information about weight loss including tips, links and other Internet resources Encouragement to use online dietary and PA self-monitoring tools Weekly email reminders sent by therapist to submit self-monitoring data	Exercise energy expenditure (kcal/week)^‡^	I: 886 ± 832 C: 803 ± 1015	I: 342 ± 945^§^ C: 63 ± 1211^§^	Body weight and waist circumference	No significant between-group differences for exercise energy expenditure
McKay et al [40], 2001	Access to website containing a personalized PA program based on baseline online assessment of PA level; PA program consisted of personalized goal setting, activity selection, scheduling PA, overcoming barriers Access to personal PA database containing additional PA-related information and PA logs with graphs of progress Provision of personalized counseling and support provided by a personal coach by means of online messages Access to peer-to-peer support groups	Access to website containing diabetes specific articles plus real-time blood glucose tracking with graphic feedback	Moderate-to-vigorous intensity exercise (min/day) Walking (min/day)	Exercise: I: 5.6 ± 6.2 C: 7.3 ± 6.2 Walking: I: 6.4 ± 6.2 C: 8.4 ± 8.4	Exercise: I: 17.6 ± 15.3 C: 18.0 ± 17.3 Walking: I: 12.5 ± 9.5 C: 16.8 ± 22.8	–	No significant between-group differences for moderate-to-vigorous intensity exercise or walking
Tate et al [35], 2001	One introductory face-to-face group weight loss session (1 hour) in which instructions regarding weight loss and increasing PA levels were given by clinical therapist Access to educational website containing information about weight loss, such as diet, exercise, self-monitoring, social support, stimulus control, and managing stress A brief 15 min face-to-face check-in with therapist every 3 months Instructions to report dietary and PA self-monitoring information weekly by means of website diary Weekly emails sent by therapist containing a behavioral weight loss lesson, personalized feedback, recommendations, reinforcements, answers to participants’ questions, and general support Access to electronic bulletin board	One introductory face-to-face group weight loss session (1 hour) in which instructions regarding weight loss and increasing PA levels were given by clinical therapist Access to educational website containing information about weight loss, such as diet, exercise, self-monitoring, social support, stimulus control, and managing stress A brief 15 min face-to-face check-in with therapist every 3 months Encouragements to use online dietary and PA self-monitoring tools	Exercise energy expenditure (kcal/week)^‡^	I: 1360 ± 1415 C: 1031 ± 981	I: 1289 ± 919 C: 1125 ± 1320	Body weight and waist circumference	No significant between-group differences for exercise energy expenditure
**Section C: comparison of two types of Internet-based physical activity interventions that differ with respect to the applied treatment procedures**							
Hageman et al [43], 2005	One initial face-to-face assessment of behavioral markers and biomarkers Three online newsletters containing individually tailored information about PA goals, benefits. and barriers to PA and self-efficacy delivered by Internet every month	One initial face-to-face assessment of behavioral markers and biomarkers Three online newsletters containing general information about PA goals, benefits, and barriers to PA and self-efficacy delivered by Internet every month	Moderate or vigorous PA (min/week) Energy expenditure (kcal/kg/day)	PA: I: 937.6 ± 616.5 C: 1228.1 ± 119.7 Expenditure: I: 28.7 ± 5.0 C: 28.9 ± 5.7	PA:^||^ I: 672.5 ± 643.9 C: 906.0 ± 775.8 Expenditure:^||^ I: 26.5 ± 5.0 C: 27.3 ± 4.6	Cardiorespiratory fitness, flexibility, body composition	No significant between-group differences for moderate or vigorous PA or energy expenditure
Rovniak et al [32], 2005	One 30 min face-to-face session providing information about walking plus modeling of 3 walking skills Specific and tailored email-based walking prescription by supervisor Immediate and precise self-monitoring of walking information by participants by means of online walking logs Weekly specific feedback by supervisor about walking performance relative to past accomplishments and normative standards sent by email	One 30 min face-to-face session only providing information about walking General email-based walking prescription by supervisor General self-monitoring of walking information by participants by means of online walking logs Weekly general feedback sent by supervisor about walking performance	Walking (min/week)^‡^	I: 17.5 ± 20.9 C: 16.4 ± 24.8	I: 74.5 ± 49.9 C: 61.2 ± 38.8	Cardiorespiratory fitness, walking speed, body mass index	No significant between-group differences for walking time
Marshall et al [34], 2003	Access to a stage-targeted PA website containing stage-based quizzes with feedback, personalized sections on goal setting, activity planning, targeted heart rates, and a PA readiness questionnaire Personalized reinforcement emails sent every 2 weeks containing stage-targeted PA information and links to study website	Stage-targeted printed booklets sent by postal mail containing PA information based on Transtheoretical Model of Behavior Change Additional printed reinforcement letters sent by postal mail every 2 weeks containing stage-targeted PA information	Total amount of PA (MET min/week) Total amount of sitting (MET min/week)	PA:^¶^ I: 2425 ± 113 C: 2413 ± 115 Sitting time:^¶^ I: 2263 ± 57 C: 2221 ± 56	PA:^¶^ I: 2433 ± 121 C: 2518 ± 115 Sitting time:^¶^ I: 2158 ± 48 C: 2150 ± 49	–	No significant between-group differences for PA and sitting time

^*^PA indicates physical activity; I, intervention group; C, control group; MET,metabolic equivalent

^†^PA outcome measures are outcomes that measure (changes in) the amount of physical activity.

^‡^Physical activity outcome variable in this study was considered a secondary outcome.

^§^Values of posttest data represent change scores (mean ± SD).

^||^Posttest data not measured directly after the intervention (1 month after sending last newsletter).

^¶^Values of pre- and posttest data represent mean ± SE.

## Discussion

The number of randomized controlled trials on the effectiveness of Internet-based physical activity interventions is limited. This review represents the best available evidence so far. Two investigators independently assessed all articles and abstracts, and consensus was reached concerning both the inclusion of the studies and the data extraction.

Three studies were identified that investigated whether an Internet-based physical activity intervention was more effective than a waiting list. Two of these studies reported a significantly greater increase in physical activity in the Internet-based intervention than in the waiting list group. However, the effect sizes, which were reported in only one of these two studies, were small, indicating that the clinical relevance remains questionable.

In four studies, two types of Internet-based intervention were compared in which the most important difference between the intervention and control groups was the amount of contact with the supervisors. Of these studies, only one reported significant differences between the two interventions with respect to change in physical activity level. However, in this study, the amount of personalized supervision was not the only difference between the intervention and control groups. As opposed to the participants from the control group, participants from the intervention group also received a bicycle ergometer and were offered peer-to-peer group contacts. Therefore, it could not be established if the increased amount of contact caused the increase in effectiveness. None of the three studies in which different types of treatment procedures of two Internet-based physical activity interventions were compared reported significant differences.

The methodological quality of the selected studies in this review varied. Only half of the 10 studies were rated as having a good methodological quality. Lack of information about blinding of the outcome assessor, no description of sample size calculation, and insufficient description of the randomization and concealment method were the most important reasons for low scores on methodological quality. This may have influenced the results of these studies since it has been shown that inadequate methodological approaches in controlled trials, particularly those representing poor allocation concealment, are associated with bias [44]. Furthermore, none of the studies applied an intention-to-treat analysis. However, a full application of the intent-to-treat model according to the definition given by Hollis and Campbell [38] may not be possible for most physical activity studies because, in most of these studies, there will be at least some subjects who drop out, refuse to complete final assessments, or change residence.

In addition, we evaluated the quality of the studies by assessing whether or not the interventions fulfilled criteria that apply to Internet-based physical activity interventions in particular, including intervention measures, process measures, and outcome measures. It was shown that in six studies the researchers used one or more theoretical models to compose the interventions. The Transtheoretical Model and the Social Cognitive Theory were the two most frequently used theories. This review could not demonstrate that theory-based physical activity interventions conducted through the Internet are more effective than non-theory-based interventions. Although there is some evidence that interventions in which these models are incorporated are effective in increasing physical activity level [45-47], other researchers still question this effectiveness [48]. Further research on the surplus value of these models in promoting complex health behavior such as physical activity is needed.

Furthermore, the results show that most of the studies used a single physical activity outcome measure, and objective measures such as activity monitors or pedometers were rarely used. In order to be able to better establish the effect of Internet-based physical activity interventions, future studies should incorporate multiple physical activity outcomes, preferably accompanied by one or two objective measures. Moreover, there is a need for more uniform physical activity outcome measures; in our review, studies reported their outcomes in time, energy expenditure, or categorical variables such as proportions of persons meeting physical activity recommendations.

On the basis of the above-mentioned results of this review, we conclude that there is indicative evidence that Internet-based physical activity interventions are more effective than a waiting list group. With respect to which components serve as the key components (ie, amount of contact or type of treatment procedure), the evidence is scanty.

Several factors may have contributed to the limited evidence of effectiveness. First, the number of eligible studies was limited. The Internet is a relatively new tool for delivering physical activity interventions. Moreover, many of the interventions that did use the Internet for program delivery did not report their outcomes in terms of changes in physical activity level, but used indirect measures such as stages of motivational readiness, weight change, heart rate, or maximal oxygen uptake. Our review included three studies in which the changes in physical activity level were considered secondary outcomes; these interventions were not primarily aimed at changing physical activity behavior. These three studies all compared two different types of Internet-based intervention.

Second, this review comprised mainly short-term physical activity interventions. Only three studies incorporated interventions of 6 months or longer. The literature suggests that long-term changes in physical activity behavior can only by accomplished by studies with long-term follow-up [18]. However, no guidelines exist regarding the optimal duration of interventions. Therefore, more research should be done to evaluate the minimal duration of physical activity interventions in order to produce long-term physical activity behavior change.

Third, the baseline physical activity levels of the participants differed, making it difficult to report on the overall effectiveness of these interventions. Moreover, four studies in this review did not report any baseline physical activity levels. Since physically active persons in general are better able to comply with physical activity interventions and maintain a healthy lifestyle than sedentary persons [49-51], incomplete or inconsistent information about baseline physical activity levels may have influenced our results.

A final limitation is the fact that the contents of the control intervention differed widely. In some studies, participants from the control group received more general or standard versions of the Internet-based physical activity intervention; in other studies, these participants received a print-based version of the intervention or were assigned to a waiting list. The exact surplus value of adding personalized supervision to an Internet-based physical activity intervention could not be established because, in most studies, in addition to this supervision, other components were added as well. The two trials that compared the Internet-based physical activity intervention with a waiting list both reported significant differences between the intervention and control groups. This may indicate that, when trying to increase people’s physical activity level, providing an Internet-based physical activity intervention is more effective than doing little or nothing. However, more studies are needed to establish this conclusion. With respect to determining the effectiveness of different components of an Internet-based physical activity intervention, more studies are needed that use appropriate research designs (ie, designs in which the only difference between the intervention and control groups is the addition of a specific component, such as providing personalized supervision).

In conclusion, the methodological quality as well as the type of physical activity outcome measure of Internet-based physical activity interventions varied. However, Internet-based physical activity interventions appear to be more effective when compared to a waiting list strategy. Whether or not adding specific components to Internet-based physical activity interventions will result in greater effectiveness compared to Internet-based interventions in which these components are missing or offered less intensely remains to be established. An important advantage of Internet-based interventions is that they can reach large numbers of people at relatively low cost. However, more cost-effectiveness studies should be done in order to establish the exact surplus value of this delivery method when compared with more traditional methods such as face-to-face sessions. Moreover, future research should properly define the control groups and incorporate both long-term as well uniform and objective physical activity outcome measures.

## Multimedia Appendix

### Criteria of Methodological Quality

1. Were the eligibility criteria specified?
2. Was the method of randomization described?
3. Was the random allocation concealed? (ie, Was the assignment generated by an independent person not responsible for determining the eligibility of the patients?)
4. Were the groups similar at baseline regarding important prognostic indicators?
5. Were both the index and the control interventions explicitly described?
6. Was the compliance or adherence with the interventions described?
7. Was the outcome assessor blinded to the interventions?
8. Was the dropout rate described and were the characteristics of the dropouts compared with the completers of the study?
9. Was a long-term follow-up measurement performed (outcomes measured ≥ 6 months after randomization)?
10. Was the timing of the outcome measurements in both groups comparable?
11. Was the sample size for each group described by means of a power calculation?
12. Did the analysis include an intention-to-treat analysis?
13. Were point estimates and measures of variability presented for the primary outcome measures?
